# Genome-Wide Characterization of the *Expansin* Gene Family in Eggplant (*Solanum melongena* L.) Reveals Its Roles in Fruit Development and Heat Stress Response

**DOI:** 10.3390/plants15070995

**Published:** 2026-03-24

**Authors:** Jiawei Pan, Aidong Zhang, Kai Xiao, Toheed Anwar, Kun Ma, Xuexia Wu

**Affiliations:** 1 Shanghai Key Laboratory of Protected Horticultural Technology, Horticultural Research Institute, Shanghai Academy of Agricultural Sciences, Shanghai 201403, China; panjiawei199703@163.com (J.P.); family20082008@sina.com (A.Z.); 15562791500@163.com (K.X.); 2 Hubei Collaborative Innovation Center for Grain Industry, Research Center of Crop Stress Resistance Technologies, Yangtze University, Jingzhou 434025, China; toheed.agric92@yahoo.com

**Keywords:** expansin, eggplant, genome-wide identification, fruit development, heat stress

## Abstract

Expansins are essential regulators of plant cell wall loosening, yet their roles in eggplant (*Solanum melongena* L.) remain poorly understood. This study performed a genome-wide analysis and identified 26 *SmEXP* genes, categorized into five evolutionary groups. All SmEXP proteins harbor characteristic DPBB_1 and Expansin_C domains. These genes are unevenly distributed across 10 chromosomes out of the 12 eggplant chromosomes, with Chromosome 8 identified as a major distribution hotspot. Synteny and selection pressure analyses suggest that segmental duplications and strong purifying selection have driven the family’s evolution. Promoter analysis revealed various cis-acting elements associated with light, phytohormones, and abiotic stress. Transcriptomic profiling showed that 14 *SmEXP* genes were significantly upregulated during the rapid fruit expansion phase (14 DAP), indicating their crucial role in fruit morphogenesis. Furthermore, some specific members (*SmEXP1*, *4*, *10*, and *13*) exhibited distinct upregulation under heat stress (38 °C and 43 °C), suggesting involvement in thermotolerance. These findings identify key expansin genes controlling eggplant development and stress response, providing targets for genetic improvement.

## 1. Introduction

Plant cell walls provide rigid structural support yet remain pliable enough to permit cell expansion during growth [1,2]. The key to this paradox is controlled wall loosening, driven chiefly by expansin proteins. Expansins were first isolated in 1992 from growing cucumber hypocotyls, where they were shown to be the direct cause of “acid growth,” the classic model for pH-dependent cell expansion [3]. Building on this seminal discovery, expansins are now recognized as a superfamily of wall-loosening proteins. They facilitate cell expansion by selectively disrupting the hydrogen bonds tethering cellulose to hemicelluloses (xyloglucan), thereby relaxing the wall without hydrolysis [4]. Beyond enabling cell expansion, expansins regulate a wide range of developmental programs and stress responses, influencing processes from root architecture and fruit ripening to adaptations under environmental challenge [5]. Structurally, canonical expansins are composed of two distinct domains: an N-terminal domain (domain I) with a double-psi beta-barrel fold (DPBB), resembling the catalytic site of glycosyl hydrolase family 45 (GH45), and a C-terminal domain (domain II) with a beta-sandwich fold implicated in polysaccharide binding [6,7,8]. Based on phylogenetic divergence and sequence conservation, the superfamily is classified into four subfamilies: *α*-expansins (EXPA), *β*-expansins (EXPB), expansin-like A (EXLA), and expansin-like B (EXLB) [9].

Genome-wide surveys have highlighted the remarkable evolutionary expansion and divergence of the expansin superfamily, showing that gene copy numbers vary significantly to meet specific developmental needs. Massive gene duplication events in the *Poaceae* have resulted in large families in monocots such as wheat (241 genes) [10], maize (88 genes) [11], and Moso bamboo (82 genes) [12]. While dicot family sizes are generally smaller and vary among species like cotton (93 genes) [13] and various cucurbits [14,15], they remain relatively conserved within the *Solanaceae*, with 38 genes identified in tomato [16], 36 genes identified in potato [17], and 52 genes identified in tobacco [18]. These specialized expansin repertoires are essential for coordinating diverse programs, ranging from cotton fiber elongation [13] to storage root formation in sweet potato [19,20]. However, despite being a globally vital *Solanaceous* vegetable, eggplant (*Solanum melongena* L.) lacks a comprehensive genome-wide inventory of its expansin family. Although the expansin family has been thoroughly characterized in related *Solanaceous* species such as tomato [16], potato [17], and tobacco [18]. This represents a significant knowledge gap, as understanding the specific composition and expression of eggplant expansins is essential for dissecting the molecular mechanisms governing its unique fruit development and stress resilience compared to its close relatives.

Comparative genomics studies have extensively examined the role of expansins in reproductive development, especially in fruit crops, where traits such as texture and size hold significant economic importance. In the *Solanaceae* family, to which eggplant belongs, expansins are critical regulators of fruit ripening and softening. Research in tomato has characterized the specific contribution of *EXPA* to the disassembly of cell wall architecture during fruit development [16,21]. In bell pepper (*Capsicum annuum* L.), a close relative of eggplant, temporal expression profiling of fruit-specific α-expansins has demonstrated their essential role in the rapid phase of cell expansion [22]. Similarly, in non-*solanaceous* fruits, genome-wide screens have identified five expansin genes in *Rubus chingii* [23] and a specific set of genes in strawberry (*Fragaria vesca*) [24], all of which show developmentally regulated expression patterns correlated with fruit expansion and softening. In netted melon (*Cucumis melo* L. var. reticulatus Naud.), a genome-wide study linked the transcriptional activation of certain expansin genes to fruit peel cracking, suggesting a direct role in reinforcing peel integrity against mechanical stress [25]. Furthermore, studies reveal complex transcriptional regulation of these genes. In banana (*Musa* spp.), MYB transcription factors modulate fruit texture by repressing specific cell wall metabolism genes, including expansins [26]. The transcription factor *VviWOX13C* in grapes promotes fruit set by directly stimulating the expression of *VviEXPA37/38/39* [27]. Likewise, in cucumber, *CsMYB36* acts as a transcriptional regulator of fruit neck elongation, a process largely driven by expansin activity [28].

Beyond development, the expansin superfamily also acts as a key interface in plant-environment interactions, enabling adaptation to various stresses. For example, in the wild peanut (*Arachis* spp.), specific isoforms are induced by environmental stress [29], while in Populus, the expression of multiple family members is altered by abscisic acid (ABA) and low-temperature stress [30]. The role of expansins in conferring drought tolerance is well-established in cereals. This is exemplified in barley, where the expansin B genes *HvEXPB5* and *HvEXPB6* are upregulated in drought-tolerant genotypes under stress [31]. Genetic evidence further links expansins to biotic stress. In Fusarium wilt-resistant *Citrullus amarus*, resistance has been mapped to loci associated with expansin activity [32] and the investigation of expansin dynamics during fungal symbiosis in *Laccaria bicolor* [33]. These findings collectively underscore that expansins are not merely growth factors but are central components of the plant’s defense and survival mechanisms.

In this study, we performed a systematic genome-wide identification of the expansin gene family in eggplant. Our study combined genomic characterization, examining chromosomal location, phylogenetic relationships, gene structure, and conserved motifs along with expression profiling during fruit wax accumulation and under heat stress. This integrated approach pinpointed key expansin genes involved in development and stress response, providing a genetic foundation for eggplant breeding.

## 2. Results

### 2.1. Genome-Wide Identification and Characterization of the SmEXP Gene Family

A systematic genomic analysis revealed the presence of 26 EXP genes in the eggplant genome. Following standard nomenclature, these genes were designated *SmEXP1* through *SmEXP26* based on their order along the chromosomes. Comprehensive physicochemical analysis revealed that the SmEXP proteins range from 223 to 350 amino acids in length and from 24.49 to 38.30 kDa in molecular weight, with SmEXP20 and SmEXP5 being the smallest and largest, respectively. The theoretical isoelectric points (pI) spanned a wide range from 4.64 (*SmEXP14*) to 10.01 (*SmEXP13*). Although the majority of SmEXPs were identified as basic proteins (pI > 7.00), seven members (*SmEXP14*, *16*, *8*, *20*, *2*, *17*, and *3*) were characterized as acidic (pI < 7.00). Except for *SmEXP11* (GRAVY = 0.012), all family members exhibited negative Grand Average of Hydropathicity (GRAVY) values, suggesting that the eggplant expansin family is predominantly hydrophilic. Furthermore, stability predictions based on the instability index (threshold < 40) classified most members as stable proteins, whereas *SmEXP5* (41.86), *SmEXP16* (42.43), *SmEXP2* (44.58), and *SmEXP13* (51.06) were predicted to be unstable (Table 1).

### 2.2. Phylogenetic Analysis of the SmEXP Gene Family

**Figure 1:**
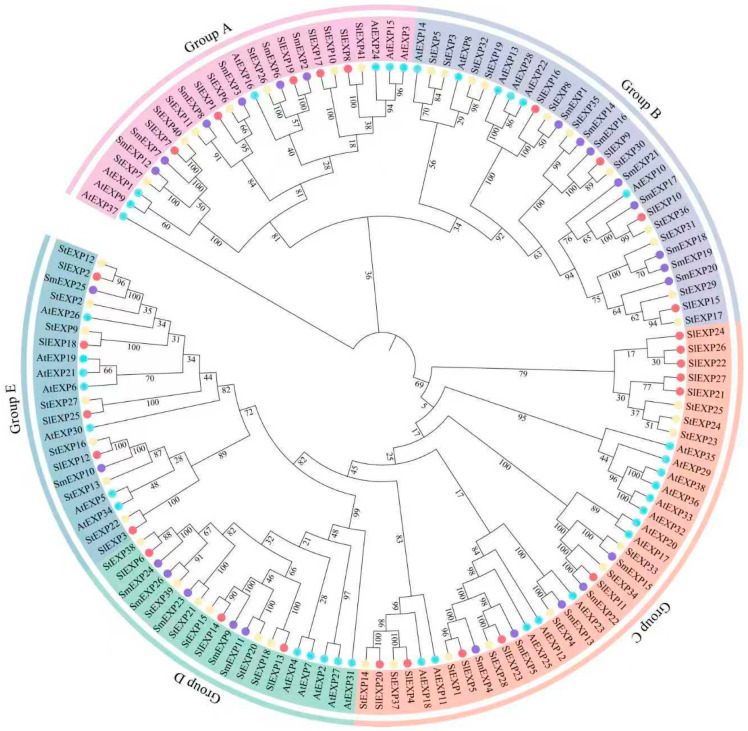
Interspecific phylogenetic analysis of EXP proteins from eggplant and three other representative plant species. The unrooted phylogenetic tree was constructed using the protein sequences of *EXP* family members from eggplant (*Solanum melongena*, *Sm*), tomato (*Solanum lycopersicum*, *Sl*), potato (*Solanum tuberosum*, *St*), and *Arabidopsis thaliana* (*At*). The 132 *EXP* proteins are clustered into five distinct evolutionary groups (Group A to Group E), which are highlighted with different background colors as indicated in the legend (top-left). Colored circles at the branch tips represent different species: purple for eggplant (*Sm*), red for tomato (*Sl*), yellow for potato (*St*), and cyan for *Arabidopsis* (*At*). The numbers at the nodes indicate bootstrap support values based on 1000 replicates.

To elucidate the evolutionary relationships among eggplant expansins, an intraspecific phylogenetic tree was constructed using the Neighbor-Joining (NJ) method (Table S1; Figure 1). Based on the phylogenetic topology and evolutionary distances, the 26 *SmEXP* members were classified into two primary clades. Clade I included 14 members (*SmEXP19*, *SmEXP18*, *SmEXP20*, *SmEXP21*, *SmEXP17*, *SmEXP1*, *SmEXP16*, *SmEXP14*, *SmEXP7*, *SmEXP12*, *SmEXP2*, *SmEXP6*, *SmEXP3*, *SmEXP8*) and Clade II had 12 members (*SmEXP15*, *SmEXP22*, *SmEXP13*, *SmEXP4*, *SmEXP5*, *SmEXP10*, *SmEXP25*, *SmEXP9*, *SmEXP11*, *SmEXP26*, *SmEXP24*, *SmEXP23*).

**Figure 2:**
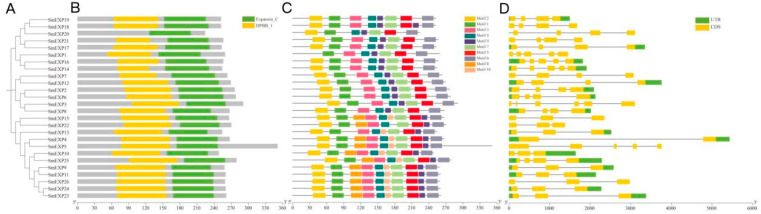
Phylogenetic relationship, conserved motifs, and gene structure of *SmEXP* genes in eggplant. (**A**) Phylogenetic tree of *SmEXP* proteins. The tree was constructed based on the full-length protein sequences of 26 *SmEXP* members using the Neighbor-Joining (NJ) method. (**B**) The conserved domain composition was identified using the Pfam database. Yellow boxes represent the DPBB_1 domain (N-terminal), and green boxes represent the Expansin_C domain (C-terminal). Grey bars indicate non-conserved protein sequences. The scale bar at the bottom denotes the protein length in amino acids (aa). (**C**) Conserved protein motifs and gene structures of *SmEXP* genes. Distribution of conserved motifs. The motifs are identified by MEME analysis and are represented by different colored boxes numbered 1–10. The scale bar at the bottom indicates the protein length (amino acids). (**D**) Exon–intron organization of *SmEXP* genes. Yellow boxes represent CDS (coding sequences); green boxes represent UTRs (untranslated regions); thin black lines represent introns. The scale bar at the bottom indicates the gene length (base pairs).

A comparative phylogenetic analysis was performed using 132 EXP proteins from eggplant (26), *Arabidopsis* (38), potato (41), and tomato (27) to elucidate the evolution of the SmEXP gene family (Figure 2). These proteins were categorized into five distinct evolutionary groups (Groups A–E). *SmEXP* members were represented across all groups: Group A (6), Group B (8), Group C (5), Group D (5), and Group E (2). Notably, the majority of *SmEXP* proteins displayed greater phylogenetic proximity to their orthologs from tomato and potato than to those from *Arabidopsis*. This clustering pattern underscores the close evolutionary affinity within the *Solanaceae* and reflects the species-specific divergence that occurred following their separation from the *Brassicaceae* lineage.

### 2.3. Conservative Domains, Motifs, and Gene Structure Analysis

To elucidate the structural conservation and evolutionary diversification of the *SmEXP* family, we performed a comprehensive analysis of their protein domains, conserved motifs, and exon–intron organizations (Figure 2).

**Figure 3:**
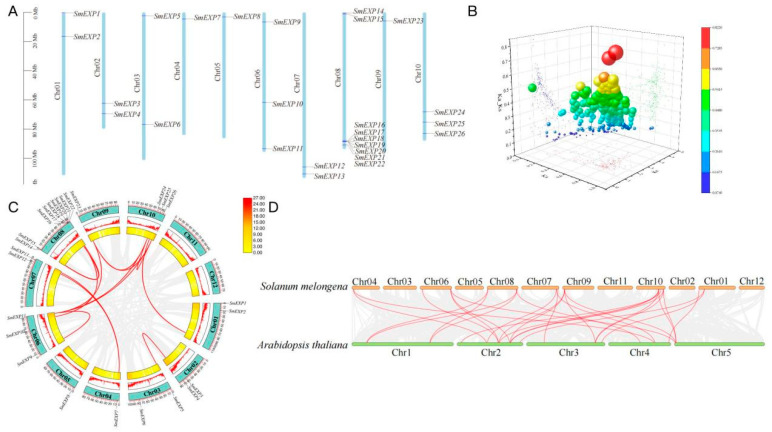
Chromosomal distribution and evolutionary analysis of *SmEXP* genes. (**A**) Chromosomal localization of *SmEXP* genes. The 26 *SmEXP* genes are mapped onto the 10 eggplant chromosomes. Chromosome numbers are indicated at the top of each bar, and the scale on the left represents the chromosomal length in Megabases (Mb). (**B**) 3D scatter plot of Ka, Ks, and Ka/Ks ratios for *SmEXP* gene pairs. The axes represent Ka, Ks, and the Ka/Ks ratio, respectively. The color and size of the spheres indicate the magnitude of the Ka/Ks value, reflecting the selection pressure during evolution. (**C**) Synteny analysis and duplication events of *SmEXP* genes in the eggplant genome. The outer circle represents the 12 chromosomes. Red lines connecting different chromosomes indicate segmental duplication gene pairs. The inner tracks represent gene density and other genomic features. (**D**) Synteny analysis of *EXP* genes between *Solanum melongena* and *Arabidopsis thaliana*. The orange and green horizontal bars represent the chromosomes of eggplant and *Arabidopsis*, respectively. Red lines highlight the orthologous EXP gene pairs between the two species, while grey lines in the background indicate collinear blocks.

Domain analysis confirmed that all 26 identified SmEXP proteins harbor the bipartite domain architecture characteristic of the expansin superfamily: the DPBB_1 (N-terminal, PF03330) and the Expansin_C (C-terminal, PF01357) domains (Figure 3). While the core catalytic and cellulose-binding regions remained highly conserved, notable variations were observed in the flanking non-conservative regions. Most SmEXP proteins, particularly those in Clade I, exhibited a typical length of 250–270 amino acids (aa). In contrast, members such as *SmEXP3* and *SmEXP5* possessed extended C-terminal tails, reaching lengths of 290–360 aa. These sequence extensions in non-conservative regions may provide structural flexibility or facilitate specific protein–protein interactions, contributing to the functional specialization of these isoforms (Figure 2A,B).

Using the MEME suite, 10 conserved motifs (Motifs 1–10) were identified across the *SmEXP* family, with their distribution patterns being highly congruent with the phylogenetic topology. Motifs 2 and 1 were found to be ubiquitous and localized at the N-terminal of nearly all members, which may contribute to the putative structural framework for expansin activity. Interestingly, Motif 8 was exclusively or primarily enriched in Clade II members, suggesting it may serve as a lineage-specific signature (Figure 2A,C).

The exon–intron organization revealed significant architectural diversity within the *SmEXP* family, providing clues regarding their genomic evolution. The majority of *SmEXP* genes contained a relatively stable structure of three to five exons. However, distinct variations were noted: *SmEXP4* underwent exon reduction (only two CDS), while *SmEXP3* exhibited increased complexity with six CDS. This variation was primarily driven by intron expansion; for instance, the significant length of *SmEXP4* is attributed to its exceptionally large introns. Such intron-mediated expansion might be associated with regulatory complexity or the presence of internal cis-elements (Figure 2A,D).

Overall, the high degree of structural similarity within specific phylogenetic sub-clades, coupled with the diversification of intron lengths and motif arrangements, suggests that the *SmEXP* family has maintained a conserved functional core while evolving diverse regulatory and structural features to adapt to complex physiological requirements in eggplant.

### 2.4. Chromosomal Mapping and Synteny Analysis

The 26 *SmEXP* genes were unevenly distributed across 10 out of the 12 eggplant chromosomes (Chr01-Chr10), with no members identified on Chr11 and Chr12. Chromosome 8 was identified as a “hotspot” for the expansin family, featuring two distinct gene clusters: *SmEXP14-15* at the proximal end and *SmEXP16-22* at the distal end (Figure 3A).

Selection pressure analysis revealed that the Ka/Ks ratios for all identified paralogous pairs were significantly less than 1 (ranging from 0.07 to 0.82). This indicates that the *SmEXP* family has been subject to a strong purifying selection during evolution, ensuring the maintenance of conserved biological functions (Figure 3B; Table S2).

Intraspecific synteny analysis identified numerous segmental duplication events, particularly involving Chromosomes 6 and 10. For instance, *SmEXP* genes on Chr 10 showed strong collinearity with members on Chr 1, 6, 9, and 10, highlighting the role of large-scale genomic rearrangements in the expansion of this family (Figure 3C). Interspecific synteny analysis between eggplant and *Arabidopsis* revealed extensive collinear blocks. A total of 29 orthologous expansin gene pairs were identified between eggplant and *Arabidopsis*. The dense orthologous connections between eggplant Chr 10 and *Arabidopsis* Chr 2 support the hypothesis that the *EXP* families in these species share a common ancestral origin and have remained relatively stable since their divergence (Figure 3D).

### 2.5. Analysis of Cis-Acting Elements in SmEXP Promoters

To understand the transcriptional regulation and potential functions of the SmEXP family, we analyzed cis-acting regulatory elements (CREs) within the 2000 bp promoter regions upstream of all 26 *SmEXP* genes. We identified a broad spectrum of CREs associated with light signaling, phytohormone responses, abiotic stress, and plant development, reflecting a sophisticated regulatory network (Table S3).

**Figure 4:**
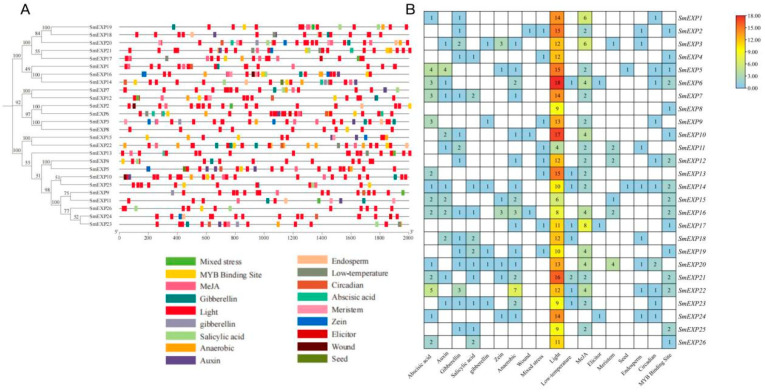
Analysis of cis-acting elements in the promoter regions of *SmEXP* genes. (**A**) Distribution of various cis-acting elements in the 2000 bp upstream promoter regions of 26 *SmEXP* genes. The Neighbor-Joining (NJ) phylogenetic tree on the left illustrates the evolutionary relationships among the members. Colored blocks on the right represent different types of *cis*-acting elements, with their specific functions indicated in the legend below. The scale bar at the bottom represents the length of the promoter sequences. (**B**) Heatmap showing the counts of identified cis-acting elements for each *SmEXP* gene. The categories include responses to hormones, abiotic/biotic stresses, and plant development. The color gradient from blue to red, along with the numerical values in each cell, represents the frequency of each motif in the respective gene promoter.

Light-responsive elements emerged as the most abundant and ubiquitous category across the entire family. These elements, including G-box, GT1-motif, and MRE, were identified in all *SmEXP* promoters, with an average of over 10 elements per gene. Notably, *SmEXP6* contained the highest density (18 elements), followed by *SmEXP10* (17 elements). The prevalence of these motifs suggests that light signaling serves as a primary environmental cue, potentially modulating expansin-mediated cell wall loosening during eggplant photomorphogenesis and daily growth cycles (Figure 4). Phytohormone-responsive elements were also extensively represented, indicating that *SmEXP* genes are integral components of multiple hormonal signaling pathways. Elements responsive to abscisic acid (ABRE), methyl jasmonate (CGTCA/TGACG-motif), salicylic acid (TCA-element), and auxin (AuxRR-core/TGA-element) were widely distributed. Specifically, MeJA-responsive elements were significantly enriched in *SmEXP17*, suggesting their involvement in jasmonate-mediated defense responses or senescence. The presence of ABREs in the majority of *SmEXP* members points to a core role in mediating growth-stress trade-offs under osmotic or ABA-regulated developmental stages. The promoters also harbored various abiotic stress-related elements, highlighting the role of *SmEXP* genes in environmental adaptation. These included anaerobic induction motifs (ARE), MYB-binding sites (MBS) involved in drought-inducibility, and low-temperature responsive (LTR) elements. Notably, *SmEXP22* possessed seven ARE motifs, implying its potential involvement in hypoxia signaling or adaptation to waterlogging stress (Figure 4). Furthermore, growth and development-specific elements were identified, providing evidence for the spatio-temporal regulation of SmEXP genes. For instance, elements associated with seed-specific regulation elements (RY-element) were identified in *SmEXP5*, *14* (Figure 4).

### 2.6. Expression Profiling of Expansin Genes Associated with Fruit Wax Accumulation

To explore the potential involvement of *SmEXP* genes in fruit surface development and wax deposition, we analyzed their expression patterns in three eggplant accessions with contrasting wax contents: 22-1 (high-wax), 30-1 (low-wax), and QPCQ (low-wax) at two critical developmental stages (14 and 22 days after pollination, DAP).

**Figure 5:**
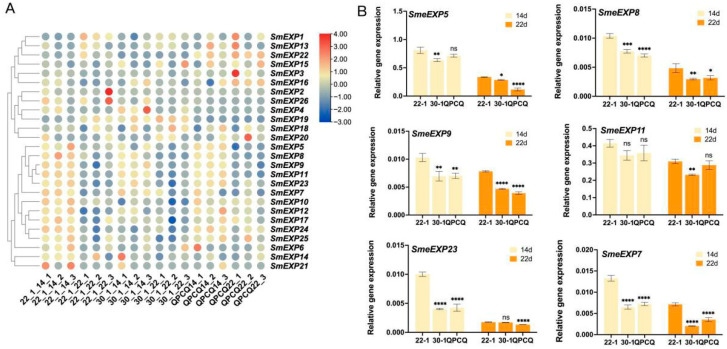
Transcriptional profiling and qPCR validation of *SmEXP* genes in eggplant materials with different wax contents. (**A**) The heatmap displays the expression levels of 26 SmEXP genes in high-wax (22-1) and low-wax (30-1 and QPCQ) eggplant accessions at 14 and 22 days after pollination (DAP). 22-14 and 22-22: 22-1 material at 14 and 22 DAP, respectively. 30-14 and 30-22: 30-1 material at 14 and 22 DAP, respectively. QPCQ-14 and QPCQ-22: QPCQ material at 14 and 22 DAP, respectively. The color scale represents the Z-score normalized FPKM values, with red indicating high expression and blue indicating low expression. Hierarchical clustering of genes is shown on the left. Each column represents one independent biological replicate. (**B**) Relative expression levels of *SmEXP5*, *SmEXP8*, *SmEXP9*, *SmEXP11*, *SmEXP23*, and *SmEXP7* in three eggplant accessions: 22-1 (high-wax), 30-1 (low-wax), and QPCQ (low-wax) at 14 and 22 DAP. Each bar represents the mean ± SD of three independent biological replicates. * *p* < 0.05, ** *p* < 0.01, *** *p* < 0.001, **** *p* < 0.0001, and ns stands for not statistically significant (Student’s *t*-test).

Specifically, 14 genes, including *SmEXP5*, *8*, *9*, *11*, *23*, *7*, *10*, *12*, *17*, *24*, *25*, *6*, *14*, and *21*, exhibited a highly synchronized expression pattern across all three eggplant accessions (22-1, 30-1, and QPCQ). The transcript levels of these genes were significantly higher at 14 DAP (the rapid expansion phase) compared to 22 DAP (and later stages), where their expression markedly decreased or became nearly undetectable (Figure 5A). This consistent trend across diverse genotypes suggests that these specific expansin genes are primarily involved in the initial rapid growth and cell wall loosening of the eggplant fruit. Their sharp decline in expression as the fruit matures (from 14 to 22/30 DAP) suggests a temporally restricted role in early fruit morphogenesis, likely facilitating the rapid increase in fruit volume before wax deposition and final maturation.

To verify the expression patterns of *SmEXP* genes associated with fruit surface development and wax deposition, six candidate genes (*SmEXP5*, *SmEXP8*, *SmEXP9*, *SmEXP11*, *SmEXP23*, and *SmEXP7*) were selected for qPCR analysis across three eggplant accessions (22-1, 30-1, and QPCQ) at two developmental stages (14 and 22 DAP). The qPCR analysis confirmed a sharp temporal decline in the expression of these genes; for all six genes, transcript levels were substantially higher at 14 DAP than at 22 DAP across all genotypes. Crucially, to address the potential link between these genes and wax accumulation, we compared their expression levels among the three accessions. At the 14 DAP stage—the critical window for both rapid fruit expansion and the initiation of wax deposition—the expression levels of *SmEXP5*, *SmEXP8*, and *SmEXP9* were significantly higher in the high-wax accession ‘22-1’ compared to the low-wax accessions ‘30-1’ and ‘QPCQ’ (Figure 5). For instance, at 14 DAP, the relative expression of *SmEXP23* in the high-wax line ‘22-1’ was approximately 2.48-fold and 2.35-fold higher than in ‘30-1’and‘QPCQ’, respectively (Figure 5B).

### 2.7. Expression Profiling of Expansin Genes Under High Temperature in Eggplant

**Figure 6:**
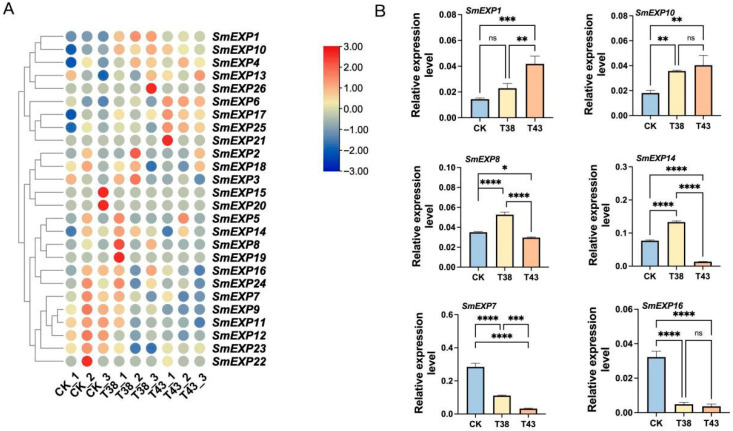
Expression profiles and qPCR validation of *SmEXP* genes in eggplant under heat stress. (**A**) The heatmap illustrates the differential expression patterns of 26 *SmEXP* genes across three conditions: CK (Control, 28 °C), T38 (38 °C), and T43 (43 °C), with three biological replicates for each treatment. The hierarchical clustering of the left group’s genes with similar expression trends. The color scale at the top right represents the Z-score normalized expression levels, where red indicates upregulation (high expression) and blue indicates downregulation (low expression). Gene names are indicated on the right side of the heatmap. (**B**) Six representative genes (*SmEXP1*, *SmEXP10*, *SmEXP8*, *SmEXP14*, *SmEXP7*, and *SmEXP16*) were selected to validate the expression patterns observed in the RNA-Seq data. CK, T38, and T43 represent the control (25 °C), 38 °C, and 43 °C heat treatments, respectively. Data are presented as the mean ± standard deviation (SD) of three independent biological replicates. * *p* < 0.05, ** *p* < 0.01, *** *p* < 0.001, **** *p* < 0.0001, and ns stands for not statistically significant (Student’s *t*-test).

We analyzed the expression patterns of all 26 *SmEXP* genes using transcriptome data from heat-stressed plants (38 °C and 43 °C) to evaluate their potential roles in abiotic stress response. The hierarchical clustering analysis revealed that the *SmEXP* genes exhibited distinct and diverse expression profiles in response to elevated temperatures (Figure 6A).

A subset of genes, including *SmEXP1*, *SmEXP10*, *SmEXP4*, and *SmEXP13*, showed significant upregulation under heat stress compared to the control (CK), suggesting that they may play crucial roles in eggplant’s thermotolerance or heat-induced cell wall remodeling. Genes such as *SmEXP5*, *SmEXP14*, *SmEXP8*, and *SmEXP19* were specifically highly expressed under 38 °C (T38) but showed reduced expression as the temperature increased to 43 °C (T43). This pattern suggests these genes might be involved in the early or moderate phase of heat acclimation. Conversely, a significant number of SmEXP members, particularly those in the bottom cluster (*SmEXP16*, *SmEXP24*, *SmEXP7*, *SmEXP9*, *SmEXP11*, *SmEXP12*, *SmEXP23*, and *SmEXP22*), were highly expressed in the control group but significantly downregulated under both T38 and T43 treatments. The downregulation of these genes under heat stress may contribute to the concurrent inhibition of vegetative growth, a common physiological response to high temperatures.

To validate the reliability of the RNA-Seq data and further characterize the response of *SmEXP* genes to high temperatures, six representative genes (*SmEXP1*, *SmEXP10*, *SmEXP8*, *SmEXP14*, *SmEXP7*, and *SmEXP16*) were selected for qPCR analysis. As shown in Figure 6B, the expression levels of *SmEXP1* and *SmEXP10* were significantly induced by heat stress. Specifically, *SmEXP1* exhibited a 2.89-fold increase at 43 °C compared to the control, suggesting its critical role in eggplant’s response to extreme heat. *SmEXP8* and *SmEXP14* showed their highest expression levels under 38 °C treatment but significantly decreased when the temperature reached 43 °C. This pattern indicates that these genes may be involved in the early sensing of heat stress or moderate heat acclimation. In contrast, the expression of *SmEXP7* and *SmEXP16* was markedly suppressed under both 38 °C and 43 °C conditions. The downregulation of these genes, which are typically associated with cell wall loosening during active growth, aligns with the physiological observation of growth inhibition under high-temperature stress. Overall, the high correlation between the qPCR results and the FPKM values from the RNA-Seq data demonstrates that the identified *SmEXP* genes are indeed responsive to heat stress and may serve as potential candidates for improving thermotolerance in eggplant.

## 3. Discussion

Expansins are key regulators of plant cell wall loosening. They facilitate growth, development, and stress responses by disrupting the non-covalent bonds between cellulose and hemicellulose [5]. Although the expansin gene family has been extensively characterized in several *Solanaceous* species, such as tomato (38 genes) [16], potato (36 genes) [17], and tobacco (52 genes) [18], a comprehensive genome-wide inventory of this family in eggplant remained elusive. In this study, we identified 26 *SmEXP* genes (Table 1), providing the first systematic insight into their evolutionary trajectory and potential functional roles in eggplant. This variation in family size across species likely reflects specific evolutionary histories and developmental requirements, a phenomenon also observed in the large-scale expansion of the expansin family in hexaploid wheat [10] and the distinct composition found in the *Brassica genus* [34].

The 26 *SmEXP* members were classified into five evolutionary groups (Group A–E), all maintaining the characteristic DPBB_1 and Expansin_C domains (Figure 2B). Our results identified Chromosome 8 as a major distribution hotspot, with segmental duplications and strong purifying selection (Ka/Ks < 1) driving the family’s evolution (Figure 3B). This aligns with findings by Yin et al. [30] in Populus, where segmental and tandem duplications followed by divergent selection were key to the superfamily’s origin. Similar structural conservation and duplication patterns have been documented in Malus × domestica [35] and pecan [36], suggesting that the expansin structural framework is highly stable across diverse plant lineages to preserve its cell wall-loosening efficacy [37].

A significant finding in this study was the peak expression of 14 *SmEXP* genes (including *SmEXP5*, *8*, *9*, *11*, *23*, *7*, *10*, *12*, *17*, *24*, *25*, *6*, *14*, and *21*) during the rapid expansion phase of eggplant fruit (14 DAP). The sharp decline at 22 DAP suggests these genes may be primarily involved in initial morphogenesis rather than playing a major role in later maturation (Figure 5A). Importantly, our qPCR validation not only confirmed this temporal trend but also revealed a clear correlation between *SmEXPs* expression and fruit wax content (Figure 5B). This is consistent with Lu et al. [16], who characterized the contribution of specific α-expansins to cell wall disassembly during tomato fruit development. The function of expansins in reproductive organ growth is conserved across species, as evidenced by their expression during maize endosperm development, where they facilitate tissue expansion [11]. In grapes, the transcription factor *VviWOX13C* directly activates *VviEXPA37/38/39* to promote fruit set [27], representing the synchronized expression patterns we observed in eggplant. The role of expansins in determining fruit texture and skin firmness is also documented in grapes [38], suggesting that the sharp decline of *SmEXP* levels we observed may be associated with the fruit ripening and softening.

*SmEXP1*, *4*, *10*, and *13* were significantly upregulated under heat stress (38 °C and 43 °C) (Figure 6A), suggesting that eggplant expansins may play an important role in the response to thermal stress, potentially by contributing to the maintenance of cell wall plasticity. This heat-responsive pattern was further validated by qPCR analysis of six representative genes (*SmEXP1*, *10*, *8*, *14*, *7*, and *16*), which showed high consistency with the RNA-Seq data (Figure 6B). A conserved role in thermotolerance is further evidenced by heterologous expression studies, such as the overexpression of the grass *PpEXP1* gene in tobacco, which conferred significantly enhanced heat stress tolerance [39]. The involvement of expansins in heat response is often linked to their regulatory cis-elements. In our study, *SmEXP* members exhibited heat-responsive expression patterns (Figure 6) comparable to the stress-responsive expansin-like B (EXLB) genes in potato, which are activated under heat and drought conditions to maintain tissue integrity [17]. Furthermore, İncili et al. [40] highlighted that expansin proteins in *Cucurbitaceae* members, such as watermelon and melon, are vital for abiotic stress responses, including extreme temperatures.

## 4. Conclusions

In conclusion, this genome-wide characterization identified 26 structurally conserved *SmEXP* genes in eggplant that evolved primarily through segmental duplications, with Chromosome 8 serving as a major evolutionary hotspot. We identified 14 core genes as the primary genetic drivers of rapid fruit wax accumulation at 14 DAP. Additionally, *SmEXP1*, *4*, *10*, and *13* as high-priority candidates for enhancing thermotolerance under extreme temperatures (38 °C and 43 °C). These findings reinforce the global role of expansins as central regulators of both plant morphogenesis [6] and environmental plasticity [12].

## 5. Materials and Methods

### 5.1. Identification of the EXP Gene Family in Eggplant

The whole-genome sequence, protein sequences, and GFF3 annotation files of eggplant (*Solanum melongena*) were retrieved and downloaded from the Sol Genomics Network (SGN, https://solgenomics.net/) based on the *S. melongena* HQ-1315 genome V2.0). To identify potential expansin members, previously reported EXP protein sequences from *Arabidopsis thaliana* were used as queries for a local BLASTP search (E-value ≤ 1 × 10^−5^) using TBtools (v2.0) software. Subsequently, the Hidden Markov Model (HMM) profiles of the expansin-specific domains, namely the DPBB_1 (PF03330) and the Expansin_C (C-terminal, PF01357) domains, were employed to scan the eggplant proteome [12]. All candidate sequences were further validated using the NCBI Conserved Domain Database (CDD) (https://www.ncbi.nlm.nih.gov/Structure/cdd/wrpsb.cgi; accessed on 6 November 2025) and the Pfam (http://pfam.xfam.org/; accessed on 6 November 2025) database to ensure the presence of essential conserved domains [41]. Redundant sequences and truncated proteins lacking typical EXP domains were manually excluded to determine the final *SmEXP* gene family members.

### 5.2. Analysis of Physicochemical Properties

The primary physicochemical characteristics of the identified SmEXP proteins were characterized using the ExPASy-ProtParam online server (https://web.expasy.org/protparam/; accessed on 8 November 2025) [42]. Key parameters, including the number of amino acids (aa), molecular weight (MW), theoretical isoelectric point (pI), instability index, and Grand Average of Hydropathicity (GRAVY), were calculated to evaluate the biochemical diversity of the SmEXP family.

### 5.3. Phylogenetic Analysis and Evolutionary Classification

To elucidate the evolutionary relationships and phylogenetic classification of the eggplant EXP gene family, a comprehensive phylogenetic tree was constructed. The protein sequences of EXP members from eggplant (*Solanum melongena*), *Arabidopsis thaliana*, tomato (*Solanum lycopersicum*), and potato (*Solanum tuberosum*) were utilized for this analysis. The reference sequences for *Arabidopsis* were retrieved from TAIR (https://www.arabidopsis.org/), while those for tomato and potato were obtained from the Sol Genomics Network (SGN) and Ensembl Plants, respectively.

Multiple sequence alignment (MSA) of all identified EXP proteins from the four species was performed using the MUSCLE (v3.8.31) within MEGA (v11.0.10). Subsequently, a phylogenetic tree was generated using the Maximum Likelihood (ML) method. To ensure the highest accuracy, the best-fit amino acid substitution model was selected based on the Bayesian Information Criterion (BIC). The robustness of the internal nodes was evaluated with 1000 bootstrap replicates. The resulting phylogenetic tree was visualized and esthetically refined using the Interactive Tree of Life (iTOL v6, https://itol.embl.de/; accessed on 12 November 2025).

### 5.4. Chromosomal Localization and Mapping

The physical mapping of *SmEXP* genes was performed to determine their distribution across the eggplant genome. The precise chromosomal coordinates, including the start and end positions of each *SmEXP* member, were retrieved from the eggplant genome GFF3 annotation file downloaded from the Sol Genomics Network (SGN).

Based on their physical locations, the genes were mapped onto the 12 eggplant chromosomes using TBtools (v2.0). To provide a clear and organized identification system, the identified genes were assigned a systematic nomenclature, *SmEXP1* through *SmEXPn*, according to their linear order from the top (short arm) to the bottom (long arm) of Chromosomes 0 to 12. Additionally, the relative lengths of the chromosomes (in Megabases, Mb) were displayed to illustrate the gene density and distribution patterns of the expansin family across the entire genome.

### 5.5. Gene Structure and Conserved Motif Analysis

The exon–intron configurations of *SmEXP* genes were determined by comparing the coding sequences (CDS) with their corresponding genomic DNA sequences and visualized via TBtools (v2.0). For conserved motif identification, the SmEXP protein sequences were submitted to the MEME Suite (Multiple Expectation Maximization for Motif Elicitation v5.5.9, http://meme-suite.org/, accessed on 16 November 2025) [43], with the maximum number of motifs set to 10. Furthermore, the conserved domains were predicted using the NCBI-CDD. The integrated visualization of gene structures, motifs, and domains was performed using the Gene Structure View tool in TBtools (v2.0).

### 5.6. Analysis of Cis-Acting Elements in the SmEXP Promoters

Promoter sequences, comprising the 2000 bp upstream of the translation initiation site (ATG), were extracted for each *SmEXP* gene from the eggplant genome using TBtools (v2.0). This analysis aimed to investigate their cis-regulatory elements and potential roles in various biological processes. These sequences were subsequently submitted to the PlantCARE database 2022 version (http://bioinformatics.psb.ugent.be/webtools/plantcare/html/, accessed on 16 November 2025) for the identification of putative cis-acting regulatory elements [44]. The identified elements were categorized based on their involvement in plant growth and development, phytohormone responses, and abiotic/biotic stress signaling.

### 5.7. Synteny and Collinearity Analysis

To investigate the expansion mechanisms and evolutionary history of the *SmEXP* gene family, both intraspecific (within *S. melongena*) and interspecific (between species) collinearity analyses were performed.

For intraspecific analysis, the whole-genome sequences and GFF3 annotation files of eggplant were analyzed using the MCScanX (Multiple Collinearity Scan toolkit) integrated in TBtools (v2.0) to identify gene duplication events, such as whole-genome duplication (WGD)/segmental duplications and tandem duplications [45].

For interspecific analysis, genomic data of eggplant and *Arabidopsis* were utilized. Orthologous gene pairs between eggplant and *Arabidopsis* were identified using the “One-Step MCScanX” plugin following the previously used method [46,47,48]. The syntenic relationships were visualized using Dual Synteny Plots and Circle Gene View to illustrate the conservation and divergence of the EXP family [49].

### 5.8. RNA-Seq Expression Pattern Analysis

This study utilized two RNA-seq datasets for expression pattern analysis. The organ-specific RNA-seq data were downloaded from the NCBI database PRJNA613773 and PRJNA72124. The first dataset included three treatment groups: control (CK, 28 °C), moderate heat stress (T38, 38 °C), and high-temperature stress (T43, 43 °C) [50]. The second dataset comprised three eggplant accessions with contrasting fruit surface characteristics: 22-1 (high-wax/bright), 30-1 (low-wax), and QPCQ (low-wax). For each accession, fruit samples were collected at two developmental stages: 14 days after pollination (14 DAP, rapid expansion phase) and 22 days after pollination (22 DAP, wax maturation phase) [51].

The expression levels of the identified *SmEXP* genes were quantified as Fragments Per Kilobase of transcript per Million mapped reads (FPKM). The IDs of *SmEXP* family members were used to blast and extract the corresponding expression values from the original transcriptome matrices. To visualize the expression patterns, heatmaps were generated using TBtools (v2.0). For better comparison of genes with different expression magnitudes, the FPKM values were Z-score normalized (row scale). Hierarchical clustering was performed based on the Euclidean distance to group genes with similar expression trends.

### 5.9. RNA Extraction and RT-qPCR Analysis

Eggplant seedlings (cv. ‘Tewangda’, provided by the Shanghai Academy of Agricultural Sciences) were cultivated in a growth chamber under a 16/8 h (light/dark) photoperiod at 28/25 °C and 60–70% relative humidity. Upon reaching the four-leaf stage, plants were subjected to heat stress at 38 °C and 43 °C for 3 h. For subsequent RNA extraction, 10 seedlings were harvested per treatment, with plants maintained at 28 °C serving as the control group [50]. Eggplant seedlings (cv. ‘Tewangda’, provided by the Shanghai Academy of Agricultural Sciences) were cultivated in a growth chamber under a 16/8 h (light/dark) photoperiod at 28/25 °C and 60–70% relative humidity. Upon reaching the four-leaf stage, plants were subjected to heat stress at 38 °C and 43 °C for 3 h. For subsequent RNA extraction, 10 seedlings were harvested per treatment, with plants maintained at 28 °C serving as the control group [51]. All samples were snap-frozen in liquid nitrogen and maintained at −80 °C for further analysis.

Total RNA from frozen leaves (heat-stressed, 38 °C and 43 °C) and fruit tissues was extracted using the Tiangen Plant RNA Kit (Tiangen, Beijing, China). RNA concentration and integrity were verified via NanoDrop 2000 (Thermo Fisher, Waltham, MA, USA) and 1.0% agarose gel electrophoresis. After DNase I treatment, 1 μg of RNA was reverse-transcribed using HiScript III RT SuperMix (Vazyme, Nanjing, China). RT-qPCR was conducted on a StepOnePlus System (Applied Biosystems, Foster City, CA, USA) with ChamQ SYBR Master Mix (Vazyme, Nanjing, China). The program involved 40 cycles (95 °C/5 s, 60 °C/30 s) followed by melting curve analysis. Relative expression levels were calculated using the 2^−ΔΔCt^ method based on three biological and technical replicates. Primers for qPCR used are listed in Table S4.

## Figures and Tables

**Table 1:** The physiochemical characteristics of expansin genes identified in eggplant.

Name	Gene ID	Number of Amino Acids	Molecular Weight	Theoretical pI	Instability Index	Aliphatic Index	Grand Average of Hydropathicity
*SmEXP1*	*Sme01G0040.1*	258	28,047.89	8.27	24.55	80.16	−0.031
*SmEXP2*	*Sme01G1638.1*	275	29,088.69	5.36	44.58	70.95	−0.074
*SmEXP3*	*Sme02G1204.1*	290	31,345.38	6.22	30.58	70.69	−0.314
*SmEXP4*	*Sme02G1842.1*	266	28,933.79	8.61	33.4	74.44	−0.103
*SmEXP5*	*Sme03G0204.1*	350	38,303.85	8.97	41.86	80.03	−0.116
*SmEXP6*	*Sme03G1594.1*	277	29,923.12	8.61	32.69	78.16	−0.082
*SmEXP7*	*Sme04G0381.1*	262	28,408.64	9.47	30.83	81.56	−0.082
*SmEXP8*	*Sme05G0270.1*	266	29,241.89	4.98	27.38	75.56	−0.22
*SmEXP9*	*Sme06G0300.1*	257	28,291.42	9.48	31	79.26	−0.025
*SmEXP10*	*Sme06G0964.1*	247	26,709.85	7.54	35.85	58.91	−0.182
*SmEXP11*	*Sme06G2719.1*	259	28,184.08	9.58	37.29	71.97	0.012
*SmEXP12*	*Sme07G1902.1*	268	28,955.9	8.72	38.26	70.93	−0.033
*SmEXP13*	*Sme07G2269.1*	253	28,475.57	10.01	51.06	67.04	−0.237
*SmEXP14*	*Sme08G0027.1*	253	27,789.18	4.64	36.34	78.66	−0.163
*SmEXP15*	*Sme08G0080.1*	265	28,773.69	9.25	24.12	64.08	−0.077
*SmEXP16*	*Sme08G1893.1*	255	27,963.35	4.97	42.43	72.27	−0.241
*SmEXP17*	*Sme08G1935.1*	252	28,389.25	5.55	22.57	79.68	−0.179
*SmEXP18*	*Sme08G1937.1*	251	27,670.07	9.15	22.84	81.59	−0.208
*SmEXP19*	*Sme08G1938.1*	251	27,956.2	8.7	27.24	78.53	−0.18
*SmEXP20*	*Sme08G1939.1*	223	24,494.86	5.28	31.36	86.59	−0.071
*SmEXP21*	*Sme08G1940.1*	255	27,744.27	7.54	37.38	95.92	0.221
*SmEXP22*	*Sme08G2142.1*	269	29,556.04	9.35	21.57	60.63	−0.009
*SmEXP23*	*Sme09G0431.1*	260	28,227.32	9.35	19.38	75	−0.015
*SmEXP24*	*Sme10G1493.1*	259	28,058.83	9.35	17.57	65.95	−0.115
*SmEXP25*	*Sme10G1880.1*	278	29,880.93	9.27	36.94	68.06	−0.08
*SmEXP26*	*Sme10G2198.1*	258	28,036.8	9.16	25.18	69.22	−0.151

## Data Availability

Data are contained within the article and Supplementary Materials.

## Supplementary Materials

The following supporting information can be downloaded at: https://www.mdpi.com/article/10.3390/plants15070995/s1, Table S1. Amino acid sequences of SmeEXP family proteins. Table S2. One-to-one orthologous relationship in eggplant. Table S3. Analysis of cis-acting regulatory elements in the promoter regions of eggplant expansin (SmEXP) genes. Table S4. qPCR primers used.
